# Gestational Diabetes Diagnosed Early in Pregnancy: Effects on Pregnancy Outcomes and Gestational Weight Gain

**DOI:** 10.1155/ije/3725182

**Published:** 2026-02-03

**Authors:** Ashton D’Souza, Hala Abdullahi, Ibrahim Ibrahim

**Affiliations:** ^1^ Department of Internal Medicine, Rochester General Hospital, Rochester, New York, USA, rochesterregional.org; ^2^ Department of Obstetrics and Gynaecology, Sidra Medicine, Doha, Qatar, sidra.org; ^3^ Department of Clinical Obstetrics & Gynaecology, Weill Cornell Medicine-Qatar, Doha, Qatar, qatar-weill.cornell.edu; ^4^ Department of Clinical Medicine, Weill Cornell Medicine-Qatar, Doha, Qatar, qatar-weill.cornell.edu

**Keywords:** gestational diabetes mellitus (GDM), gestational weight gain (GWG), high-risk pregnancy

## Abstract

**Background:**

The diagnosis and management of gestational diabetes mellitus (GDM) before 24 weeks of gestation are controversial topics. While some retrospective studies have shown that early diagnosis of GDM significantly impacts pregnancy outcomes, recent randomized controlled trials have found no benefit. This disparity in findings raises essential questions about the optimal timing of GDM diagnosis and its potential impact on maternal and neonatal outcomes.

**Objective:**

We aimed to compare pregnancy outcomes between women with GDM diagnosed early and those diagnosed during routine second‐trimester screening.

**Methods:**

This retrospective cohort study compared outcomes between women with early GDM diagnosed at < 14 weeks and those with GDM diagnosed at 24–28 weeks. Maternal and neonatal outcomes and the need for pharmacotherapy were compared using appropriate statistical tests.

**Results:**

Of 437 women with GDM, 113 (25.9%) were diagnosed early, and 324 (74.1%) were diagnosed in the second trimester. Women diagnosed early had a higher prepregnancy BMI and gained less weight during pregnancy compared to those diagnosed later (*p* < 0.05). However, maternal and neonatal outcomes and the need for pharmacotherapy did not significantly differ between the groups.

**Conclusion:**

An early diagnosis of GDM before 14 weeks, despite a higher BMI, was associated with less gestational weight gain but did not lead to significant differences in pregnancy outcomes or mode of treatment compared to later diagnosis.

## 1. Introduction

Gestational diabetes mellitus (GDM) as a pregnancy complication is significant, and its prevalence is rising with increasing rates of obesity among women [1]. Defined as “any degree of glucose intolerance with onset or first recognition during pregnancy” [2], GDM poses risks for various adverse maternal and neonatal outcomes [3].

Recent studies indicate that β‐cell dysfunction, changes in maternal insulin sensitivity, and accelerated fetal growth can be seen starting from 20 weeks of gestation in women who are subsequently diagnosed with GDM [4–7]. Despite these early indicators, the management of GDM diagnosed at this stage, often termed “early GDM,” remains debated. Women with early GDM have been found to experience higher perinatal mortality and a positive correlation with adverse pregnancy outcomes compared to those diagnosed later [8]. High fasting blood glucose (FBG) levels may indicate a higher risk of developing GDM and related complications [9–12]. However, the American Diabetes Association guidelines do not recommend screening for GDM before 24–28 weeks of pregnancy [13, 14].

The merits of early GDM screening are contentious. While early detection might theoretically reduce fetal exposure to hyperglycemia and improve outcomes, recent randomized controlled trials have shown that early screening and treatment do not significantly improve, or only modestly improve, maternal and neonatal outcomes compared to standard screening [15–18]. Moreover, the overdiagnosis associated with early screening can have multiple downstream effects, including unnecessary interventions such as labor induction and cesarean sections, increased healthcare costs, and psychological distress for women [19].

Given the high prevalence of GDM in Qatar, estimated at 31.6% [20], evaluating the effectiveness of early diagnosis versus standard second‐trimester diagnosis in improving maternal and neonatal outcomes in high‐risk pregnancies is crucial. This study primarily aims to compare outcomes in women diagnosed with GDM early in pregnancy with those diagnosed during routine second‐trimester screening using a routine oral glucose tolerance test (OGTT). By examining these effects, we seek to provide valuable clinical evidence to guide the management of GDM in Qatar.

## 2. Methods

### 2.1. Study Design

This study utilized a retrospective cohort approach and was carried out at Sidra Medicine in Doha, Qatar. At the initial antenatal visit (within 14 weeks of pregnancy), pregnant women underwent screening with FBG and HbA1c tests to identify undiagnosed Type 2 diabetes (T2DM) and distinguish it from pregnancy‐related glucose intolerance. Based on FBG results and following the World Health Organization (WHO) and International Association of Diabetes and Pregnancy Study Groups (IADPSG) criteria [20], women were categorized as having overt or preexisting T2DM (≥ 7.0 mmol/L) or GDM (5.1–6.9 mmol/L). If FBG was below 5.1 mmol/L, a 75‐g OGTT was scheduled between 24 and 28 weeks of gestation. GDM diagnosis was established if any of the following thresholds were met: FBG 5.1–6.9 mmol/L, 1‐h glucose ≥ 10.0 mmol/L, or 2‐h glucose 8.5–11.0 mmol/L [21]. The “early GDM” group included women with GDM detected before 14 weeks, while the “standard GDM” group included those identified during routine OGTT screening at 24–28 weeks of gestation.

Pregnant women who received prenatal care at Sidra Medicine were diagnosed with GDM, and those who delivered at or beyond 20 weeks of gestation were enrolled in the study. Exclusion criteria encompassed women with known or newly identified T2DM, prior bariatric surgery, and multiple pregnancies. Individuals with incomplete delivery outcome data were not considered, nor were those whose infants had congenital anomalies, to reduce ascertainment bias, given the hospital’s status as a tertiary referral center for such cases. We reviewed the electronic medical records of women who met the inclusion criteria and delivered between January 2019 and December 2020 to assess maternal and neonatal outcomes. Participants were stratified into two groups: early GDM and standard GDM.

### 2.2. Data Collection

Demographic, laboratory, clinical, and historical data were extracted from the Cerner electronic health records system at Sidra Medicine, with no reliance on paper records. The Institutional Review Board at Sidra Medicine approved the study under expedited review (IRB #1904345). All data were anonymized and coded for patient confidentiality. Data collected included maternal age, nationality, gravidity, parity, GDM treatment type, FBG or OGTT results, prepregnancy weight, and final recorded weight before delivery. Amniotic fluid index (AFI) measurements were obtained from third‐trimester ultrasound reports within Cerner.

### 2.3. Outcomes

Maternal outcomes examined included gestational age at delivery, mode of delivery, pregnancy‐induced hypertension (PIH), preeclampsia, preterm labor, labor induction, polyhydramnios, and oligohydramnios. Neonatal outcomes analyzed included type of birth (live birth, stillbirth, or neonatal death), birth weight, neonatal hypoglycemia, jaundice, shoulder dystocia, respiratory distress, and neonatal intensive care unit (NICU) admission. While some outcomes may be less directly influenced by an early GDM diagnosis, including them provides a more thorough evaluation of the potential effects of timing and may help identify subtle or unexpected associations that could impact clinical management strategies.

### 2.4. Definitions

Prepregnancy weight referred to the most recent documented weight before conception or, if unavailable, the weight at the initial prenatal appointment. If neither measurement was available, the data were marked as missing. Weekly gestational weight gain (GWG) was determined by subtracting prepregnancy weight from delivery weight and dividing the difference by the gestational age at delivery in weeks. PIH was classified as “a systolic blood pressure ≥ 140 mmHg or a diastolic pressure ≥ 90 mmHg or both on two occasions at least four hours apart after 20 weeks of gestation in a woman with previously normal blood pressure” [22]. Preeclampsia was defined as PIH combined with at least one of the following: proteinuria, pulmonary edema, thrombocytopenia, impaired liver function, renal insufficiency, or neurological symptoms [22]. Oligohydramnios was defined as an AFI ≤ 5.0 cm and polyhydramnios as an AFI ≥ 24.0 cm [23]. Birth weight percentiles were calculated using an online tool based on INTERGROWTH‐21^st^ data [24] and the Hadlock fetal weight model [25]. Macrosomia was defined as a birth weight of ≥ 4000 g, small for gestational age (SGA) as birth weight below the 10th percentile, large for gestational age (LGA) as birth weight above the 90th percentile, and preterm labor as delivery between 20 and 37 weeks of gestation. Neonatal hypoglycemia was defined as a blood glucose level below 2.6 mmol/L in newborns [26].

### 2.5. Statistical Analysis

Data analysis was performed using Microsoft Excel (Version 16.69) and SPSS Statistics (Version 29.0, IBM). Categorical data were presented as frequencies and percentages, while continuous data were summarized as means with standard deviations. The two‐sample *t*‐test was applied to continuous variables, and the chi‐square test was used for categorical variables. A significance threshold was set at *p* < 0.05.

## 3. Results

A total of 437 women with GDM were included in the study. Of these, 113 (25.9%) were diagnosed early in pregnancy before 14 weeks of gestation, and 324 (74.1%) were diagnosed by OGTT between 24 and 28 weeks of gestation. Baseline characteristics of the study cohort are depicted in Table 1. The age and ethnic composition of the two groups were similar. Women diagnosed with early GDM had a higher prepregnancy BMI (27.2 ± 4.9 vs. 26.1 ± 4.5 kg/m^2^, *p* = 0.035) and a higher rate of obesity (28.4% vs. 17.7%, *p* = 0.021). This group also experienced significantly less weight gain during pregnancy (0.29 ± 0.15 vs. 0.34 ± 0.18 kg/week, *p* = 0.012) than the standard GDM group.

**TABLE 1:** Comparison of baseline characteristics between the two groups.

	Early (*n* = 113)	Standard (*n* = 324)	*p* value
Age	32.3 ± 5.5	31.6 ± 4.8	0.153
Prepregnancy weight	69.7 ± 13.2 (*n* = 102)	67.9 ± 12.9 (*n* = 300)	0.216
BMI	27.2 ± 4.9 (*n* = 102)	26.1 ± 4.5 (*n* = 299)	0.035
Gestational weight gain	0.29 ± 0.15 (*n* = 102)	0.34 ± 0.18 (*n* = 300)	0.012
Obesity	29 (28.4%)	53 (17.7%)	0.021
Qatari	63 (55.8%)	199 (61.4%)	0.290

Table 2 compares maternal outcomes between women with early GDM and those with standard GDM. Both groups delivered at a mean gestational age of 38.3 weeks. There was no significant difference in the proportion of women requiring pharmacotherapy between the two groups (35.4% vs. 29.0%, *p* = 0.205). Rates of hypertensive disorders in pregnancy, preterm labor, and the need for labor induction were similar. In addition, the rate of C‐sections was not significantly different between the early GDM cohort (44.2%) and the standard GDM cohort (41.0%, *p* = 0.553). Other maternal outcomes were also not different between the groups.

**TABLE 2:** Incidence of maternal and neonatal outcomes in the two groups.

	Early (*n* = 113)	Standard (*n* = 324)	*p* value
Gestational age	38.3 ± 1.6	38.3 ± 1.2	0.958
Birth weight	3157.2 ± 558.7	3194.4 ± 414.0	0.518
Weight percentile	60.6 ± 28.5	61.8 ± 26.6	0.692
Duration of NICU admission	19.1 ± 26.3 (*n* = 9)	8.7 ± 12.2 (*n* = 27)	0.280
Treatment	40 (35.4%)	94 (29.0%)	0.205
PIH	2 (1.8%)	6 (1.9%)	1.000
Preeclampsia	1 (0.9%)	5 (1.5%)	1.000
Induction of labor	6 (5.3%)	13 (4.0%)	0.594
Preterm labor	3 (2.7%)	9 (2.8%)	1.000
C‐Section	50 (44.2%)	133 (41.0%)	0.553
Primary C‐section	30 (26.5%)	92 (28.4%)	0.706
Emergency C‐section	9 (8.0%)	21 (6.5%)	0.591
Elective C‐section	9 (8.0%)	17 (5.2%)	0.293
Assisted vaginal delivery	10 (8.8%)	36 (11.1%)	0.500
Polyhydramnios	3 (2.7%)	9 (2.8%)	1.000
Oligohydramnios	2 (1.8%)	9 (2.8%)	0.736
LGA	25 (22.1%)	67 (20.7%)	0.746
SGA	8 (7.1%)	10 (3.1%)	0.095
Macrosomia	6 (5.3%)	7 (2.2%)	0.108
Stillbirth	1 (0.9%)	0 (0.0%)	0.259
NICU admission	9 (8.0%)	29 (9.0%)	0.749
Neonatal hypoglycemia	2 (1.8%)	6 (1.9%)	1.000
Neonatal jaundice	1 (0.9%)	1 (0.3%)	0.451
Shoulder dystocia	2 (1.8%)	1 (0.3%)	0.165
Respiratory distress	5 (4.4%)	13 (4.0%)	0.789

Abbreviations: LGA, large for gestational age; PIH, pregnancy‐induced hypertension; SGA, small for gestational age.

Neonatal outcomes are detailed in Table 2. Neonates born to women in the early GDM cohort had a similar birth weight percentile (60.6 ± 28.5) compared to those in the standard GDM cohort (61.8 ± 26.6, *p* = 0.692). Although slightly higher in the early GDM group, there was no significant difference in SGA, LGA, or macrosomia rates between the two groups. The incidence of other adverse neonatal outcomes, such as respiratory distress, shoulder dystocia, and NICU admission, did not differ significantly between the two groups.

## 4. Discussion

In this study involving 437 women with GDM, 25.9% were diagnosed early, before 14 weeks of gestation. Women diagnosed early in pregnancy had a higher prepregnancy BMI and gained less weight compared to those diagnosed later. Despite these differences, maternal and neonatal outcomes, as well as the need for pharmacotherapy, did not differ significantly between the groups.

This study aimed to evaluate whether early diagnosis of GDM, specifically before 14 weeks of gestation, is associated with better outcomes in pregnancy and a greater need for pharmacological treatment. It is often argued that diagnosing GDM at 24–28 weeks might be too late, given that alterations in glucose metabolism can occur earlier in pregnancy [4–7]. Our findings indicate that early diagnosis of GDM did not significantly improve pregnancy outcomes despite the higher baseline BMI and increased obesity observed in this group. This cohort also gained less weight during pregnancy compared to those diagnosed later, which corroborates findings in several studies that have compared outcomes between early‐diagnosed GDM and later‐diagnosed GDM [16, 27–29]. Early dietary and pharmacologic intervention, enabled by early detection, may have minimized the risks associated with these women’s higher initial obstetric risk. The extended time for adapting to the GDM diagnosis, a longer treatment period, and more frequent follow‐ups might have contributed to these outcomes. In addition, an extended duration of GDM may have enabled greater engagement in lifestyle interventions, resulting in less GWG. While early diagnosis may offer benefits through improved continuity of care and education and enhancing compliance with management, we do not have data on adherence to these interventions.

Elevated FBG early in pregnancy is associated with an increased risk of adverse outcomes [9–11], suggesting that FBG serves as a practical initial screening tool due to its simplicity and cost‐effectiveness compared to OGTT. The proportion of women managed with diet alone versus metformin was comparable between groups, indicating that the timing of diagnosis did not affect clinical thresholds or treatment approach, as the rates of pharmacotherapy were similar. This contradicts the hypothesis that early GDM may reflect a more advanced state in the pathogenesis of diabetes.

Importantly, our maternal and neonatal outcomes analysis showed no significant differences between the early GDM and standard GDM groups. These results contradict those of previous studies by supporting early screening and treatment of GDM [8, 30, 31]; the diagnosis and management of GDM before 24 weeks do not appear to significantly impact pregnancy outcomes. These findings also contrast with the literature, which suggests increased complications and a greater need for insulin and/or metformin treatment compared with standard practice, despite early screening and treatment of GDM [27, 29, 32, 33]. However, recent studies, including multiple randomized clinical trials, have found that early screening and intervention do not improve pregnancy outcomes in GDM, findings that align with this study [15, 16, 18, 34–36].

### 4.1. Limitations

The study has several limitations. As a retrospective observational study, the timing of diagnosis was not randomized across the two groups, which could have introduced potential selection bias and limited the generalizability of the findings. Unexamined factors may have influenced outcomes, including prior GDM history, glycemic control, and early referral to secondary care. The study lacks data on patient adherence to recommended treatments and lifestyle changes, which are crucial for assessing the effectiveness of early versus standard GDM management.

### 4.2. Future Directions

Further research is needed to explore the factors contributing to the observed nonsignificant difference in outcomes between early and standard diagnosis of GDM. The comparable outcomes could be explained by effective management approaches used in both early and standard GDM cases. In addition, our study underscores the importance of individualized care that considers factors beyond the timing of GDM diagnosis, such as prepregnancy BMI, GWG, and the degree of hyperglycemia.

## 5. Conclusion

Women with early‐diagnosed GDM had a higher prepregnancy weight but did not experience worse maternal or neonatal outcomes compared to those diagnosed in the second trimester. Despite early diagnosis, the lack of adverse outcomes may be due to early dietary intervention and reduced GWG. Our findings suggest that although the early detection of GDM leads to reduced GWG, it is not associated with significant improvement in pregnancy outcomes. The benefits of early GDM screening and treatment, especially in high‐risk populations, need to be further examined.

## Author Contributions

Conceptualization and design: Ashton D’Souza, Hala Abdullahi, and Ibrahim Ibrahim. Data analysis: Ashton D’Souza. Data interpretation: Ashton D’Souza, Hala Abdullahi, and Ibrahim Ibrahim. First draft of the manuscript: Ashton D’Souza. Critical revision of the manuscript: Ashton D’Souza, Hala Abdullahi, and Ibrahim Ibrahim.

## Funding

This study was supported by the Weill Cornell Medicine‐Qatar, which funded the purchase of the SPSS Statistics software license used for data analysis. The Qatar National Library funded the open‐access publication fees of this study.

## Disclosure

All authors have read and approved the final version of the manuscript.

## Ethics Statement

This study was approved by the Institutional Review Board (IRB) at Sidra Medicine and Weill Cornell Medicine, Qatar (IRB#1904345). Due to the retrospective nature of the study, no consent was required. The study followed the ethical principles for medical research mentioned in the Declaration of Helsinki, as revised in 2013.

## Consent

Please see the Ethics Statement.

## Conflicts of Interest

The authors declare no conflicts of interest.

## Data Availability

The data that support the findings of this study are available from the corresponding author upon reasonable request.
